
JOURNAL INFORMATION
==============================
NLM Title Abbreviation: Intensive Care Med Exp
Journal ID: Intensive Care Med Exp
NLM Journal ID: 101645149

Intensive Care Medicine Experimental

EISSN: 2197-425X
Publisher: Springer

ARTICLE INFORMATION
==============================
PMCID: PMC5593804
PMID: 28895108
DOI: 10.1186/s40635-017-0149-y
Article ID: 149
Article version: 1
Subjects: Meeting Abstracts

Sepsis 2017 Paris

Paris, France. September 11–13, 2017

Electronic publication date: 2017 Sep 11
Publication date: 2017 Sep
Volume: 5
Issue: Suppl 1
Issue sponsor: Publication of this supplement was funded by the International Sepsis Forum.
Electronic Location ID: 37
Copyright: © The Author(s). 2017
License: Open AccessThis article is distributed under the terms of the Creative Commons Attribution 4.0 International License (http://creativecommons.org/licenses/by/4.0/), which permits unrestricted use, distribution, and reproduction in any medium, provided you give appropriate credit to the original author(s) and the source, provide a link to the Creative Commons license, and indicate if changes were made.
License URL: https://creativecommons.org/licenses/by/4.0/

Conference name: Sepsis 2017
Conference Acronym: Sepsis 2017
Conference location: Paris, France
Conference date: September 11 - 13, 2017
issue-copyright-statement: © The Author(s) 2017

==============================
“Download to read this full article”

Publisher’s Note

Springer Nature remains neutral with regard to jurisdictional claims in published maps and institutional affiliations.

